# Preparedness of primary and secondary health facilities in India to address major noncommunicable diseases: results of a National Noncommunicable Disease Monitoring Survey (NNMS)

**DOI:** 10.1186/s12913-021-06530-0

**Published:** 2021-07-31

**Authors:** Anand Krishnan, Prashant Mathur, Vaitheeswaran Kulothungan, Harshal Ramesh Salve, Sravya Leburu, Ritvik Amarchand, Baridalyne Nongkynrih, Himanshu Kumar Chaturvedi, P. Ganeshkumar, Vinay Urs K S, Avula Laxmaiah, Manjit Boruah, Sanjeev Kumar, Binod Kumar Patro, Pankaja Ravi Raghav, Prabu Rajkumar, P. Sankara Sarma, Rinku Sharma, Muralidhar Tambe, N. Arlappa, Tulika Goswami Mahanta, Pranab Jyoti Bhuyan, Rajnish P. Joshi, Abhijit P. Pakhare, Abhiruchi Galhotra, Dewesh Kumar, Binod Kumar Behera, Roshan K. Topno, Manoj Kumar Gupta, Neeti Rustagi, Atulkumar V. Trivedi, K. R. Thankappan, Sonia Gupta, Suneela Garg, Sangita Chandrakant Shelke, ICMR-NNMS investigator group, Anand Krishnan, Prashant Mathur, Vaitheeswaran Kulothungan, Harshal Ramesh Salve, Sravya Leburu, Ritvik Amarchand, Baridalyne Nongkynrih, Himanshu Kumar Chaturvedi, P. Ganeshkumar, Vinay Urs K S, Avula Laxmaiah, Manjit Boruah, Sanjeev Kumar, Binod Kumar Patro, Pankaja Ravi Raghav, Prabu Rajkumar, P. Sankara Sarma, Rinku Sharma, Muralidhar Tambe, Co-investigators, N. Arlappa, Tulika Goswami Mahanta, Rajnish P. Joshi, Abhijit P. Pakhare, Binod Kumar Behera, Manoj Kumar Gupta, Neeti Rustagi, K. R. Thankappan, Sonia Gupta, Sangita Chandrakant Shelke, Collaborators, Pranab Jyoti Bhuyan, Abhiruchi Galhotra, Dewesh Kumar, Roshan K. Topno, Atulkumar V. Trivedi, Suneela Garg

**Affiliations:** 1 Centre for Community Medicine, All India Institute of Medical Sciences, New Delhi, India; 2 Indian Council Medical Research – National Centre for Disease Informatics and Research, Nirmal Bhawan-ICMR Complex (II Floor), Poojanahalli, Kannamangala Post, Bengaluru, Karnataka 562 110 India; 3 Indian Council Medical Research - National Institute of Medical Statistics, New Delhi, India; 4 Indian Council Medical Research - National Institute of Epidemiology, Chennai, Tamil Nadu India; 5 Division of Public Health Nutrition, Indian Council Medical Research - National Institute of Nutrition, Hyderabad, Telangana India; 6 Department of Community Medicine, Assam Medical College, Dibrugarh, Assam India; 7 Department of Community and Family Medicine, All India Institute of Medical Sciences, Bhopal, Madhya Pradesh India; 8 Department of Community and Family Medicine, All India Institute of Medical Sciences, Bhubaneshwar, Odisha India; 9 Department of Community Medicine and Family Medicine, All India Institute of Medical Sciences, Jodhpur, Rajasthan India; 10 Achutha Menon Centre for Health Science Studies, Sree Chitra Tirunal Institute for Medical Sciences and Technology, Thiruvananthapuram, Kerala India; 11 Centre for Noncommunicable Diseases, National Centre for Disease Control, Directorate General of Health Services, New Delhi, India; 12 Department of Community Medicine, B J Govt. Medical College, Pune, Maharashtra India; 13 Department of Community Medicine / Prevention & Social Medicine, Tezpur Medical College, Tezpur, Assam India; 14 Regional Director Office, Ministry of Health and Family Welfare, Guwahati, Assam India; 15 Department of General Medicine, All India Institute of Medical Sciences, Bhopal, Madhya Pradesh India; 16 Department of Community and Family Medicine, All India Institute of Medical Sciences, Raipur, Chattisgarh India; 17 Department of Preventive and Social Medicine, Rajendra Institute of Medical Sciences, Ranchi, Jharkhand India; 18 Department of Epidemiology, Indian Council Medical Research - Rajendra Memorial Research Institute of Medical Sciences, Patna, Bihar India; 19 Department of Community Medicine, Government Medical College, Bhavnagar, Gujarat India; 20 Department of Public Health and Community Medicine, Central University Kerala, Kasaragod, Kerala India; 21 Department of Community Medicine, Maulana Azad Medical College and Associated Hospitals, New Delhi, India

**Keywords:** Health systems, Human resources, Medicines, Noncommunicable diseases, Primary health care, Technologies

## Abstract

**Background:**

The monitoring framework for evaluating health system response to noncommunicable diseases (NCDs) include indicators to assess availability of affordable basic technologies and essential medicines to treat them in both public and private primary care facilities. The Government of India launched the National Program for Prevention and Control of Cancer, Diabetes, Cardiovascular diseases and Stroke (NPCDCS) in 2010 to strengthen health systems. We assessed availability of trained human resources, essential medicines and technologies for diabetes, cardiovascular and chronic respiratory diseases as one of the components of the National Noncommunicable Disease Monitoring Survey (NNMS - 2017-18).

**Methods:**

NNMS was a cross-sectional survey. Health facility survey component covered three public [Primary health centre (PHC), Community health centre (CHC) and District hospital (DH)] and one private primary in each of the 600 primary sampling units (PSUs) selected by stratified multistage random sampling to be nationally representative. Survey teams interviewed medical officers, laboratory technicians, and pharmacists using an adapted World Health Organization (WHO) – Service Availability and Readiness Assessment (SARA) tool on handhelds with Open Data Kit (ODK) technology. List of essential medicines and technology was according to WHO - Package of Essential Medicines and Technologies for NCDs (PEN) and NPCDCS guidelines for primary and secondary facilities, respectively. Availability was defined as reported to be generally available within facility premises.

**Results:**

Total of 537 public and 512 private primary facilities, 386 CHCs and 334 DHs across India were covered. NPCDCS was being implemented in 72.8% of CHCs and 86.8% of DHs. All essential technologies and medicines available to manage three NCDs in primary care varied between 1.1% (95% CI; 0.3–3.3) in rural public to 9.0% (95% CI; 6.2–13.0) in urban private facilities. In NPCDCS implementing districts, 0.4% of CHCs and 14.5% of the DHs were fully equipped. DHs were well staffed, CHCs had deficits in physiotherapist and specialist positions, whereas PHCs reported shortage of nurse-midwives and health assistants. Training under NPCDCS was uniformly poor across all facilities.

**Conclusion:**

Both private and public primary care facilities and public secondary facilities are currently not adequately prepared to comprehensively address the burden of NCDs in India.

**Supplementary Information:**

The online version contains supplementary material available at 10.1186/s12913-021-06530-0.

## Background

One of the objectives of the World Health Organization (WHO) Global Action Plan (2013–2020) for prevention and control of noncommunicable diseases (NCDs) was to strengthen and reorient health systems to address four major NCDs - cardiovascular disease, cancer, chronic respiratory disease, and diabetes by increasing access to affordable and effective medicines as well as technologies and improving availability of skilled workforce [1]. As a part of their NCD monitoring framework, both WHO [1] and Government of India (GoI) [2] have identified two targets for assessing health system response — 50% coverage of drug therapy and counselling; and making affordable basic technologies and essential medicines required to treat major noncommunicable diseases available in 80% of public and private primary care facilities [1, 2].

The WHO has defined a package of essential medicines and technologies for NCDs (PEN) as minimum requirements to manage NCDs at primary care, which excludes cancer [3]. However, countries are still struggling to strengthen their primary health care to address NCDs. An assessment of preparedness of primary care facilities for cardiovascular disease in Madhya Pradesh, India showed critical gaps in human resource and laboratory services followed by availability of essential medicines, equipment and related supplies [4]. The medicine-availability studies conducted between 2008 and 2015 have shown lower availability of medicines in public sector facilities compared to the private sector [5, 6]. No information is available from private sector in India on their preparedness to deal with NCDs, despite studies showing that almost 66% of the hospital admissions due to NCDs occur in private sector [7].

The health care infrastructure in India has been developed as a three-tier system and is based on the following population norms – subcentre (one for 3000–5000) population; Primary Health Centre (PHC) (one for 20,000–30,000 population) and Community Health Centre (CHC) (one for every 80,000 – 1,20,000 population). Above this is the District Hospital (DH) with around 200 beds serving about 10,00,000 - 20,00,000 population [8].

Recognizing the problem of NCDs, the Ministry of Health and Family Welfare (MoHFW), GoI launched the National Programme for Prevention and Control of Cancer, Diabetes, Cardio-vascular Diseases and Stroke in 2010. Its primary focus is to strengthen health systems by building capacity at all levels of public health system including training human resources for prevention and control of common NCDs [9]. Also, provisions have been made to provide free diagnostic facilities and medicines for patients attending the NCD clinics at the DHs and CHCs. Until March 2017, 388 NCD clinics at district hospitals (out of 756 DHs) and 2115 at CHCs (out of 5685) had been established in the country [10]. The program is still evolving and being scaled up.

The monitoring framework for this program has not been fully operationalized. No national level health facility preparedness survey to tackle NCDs has been carried out in the country [11]. Addressing NCDs requires sustainable approaches integrated with health promotion and prevention measures, workforce availability and training as well as effective monitoring systems. Recognizing this gap, the National NCD Monitoring Survey (NNMS) was implemented by the Indian Council of Medical Research (ICMR) - National Centre for Disease Informatics and Research (NCDIR) with technical support from the All India Institute of Medical Sciences (AIIMS), New Delhi along with its implementation through nine other partner agencies. The primary objective of the survey was to generate national data on NCD risk factors in adults (18–69 years) and adolescents (15–17 years), health seeking behaviours and national health system response to NCDs. The results of the adult data are published separately [12]. This paper describes the results of the health facility preparedness assessment conducted as a part of this survey. NNMS adapted global tools to understand the health systems performance and readiness related to infrastructure, service delivery, workforce and availability of essential medicines and technologies for NCDs. The survey assessed both primary (public and private) and secondary (public only) health facilities for their preparedness in dealing with diabetes mellitus (DM), cardiovascular (CVD) and chronic respiratory diseases (CRD).

## Methods

The National NCD Monitoring Survey was a national level community-based cross-sectional survey conducted during October 2017 to April 2018. The survey adopted a stratified multistage geographically clustered, probability-based sampling design using the 2011 Census as its sampling frame to cover the age groups between 15 and 69 years, including a sub-set of adolescents between 15 and 17 years. The target study population was divided into four subgroups of urban and rural by gender (men and women) [12]. The national representative sample size of 12,000 households (equally allocated to urban and rural areas) was arrived to estimate NCD risk factors among adults (18–69 years) and adolescents (15–17 years). To arrive at this desired national sample, the country was divided into 10 contiguous zones each with approximately 60 clusters. The 300 primary sampling units (PSUs) from the rural sampling frame and 300 wards from the urban sampling frame were selected using the probability proportional to population size (PPS) method [12]. A total of 600 PSUs (300 from urban and 300 from rural) were required considering 20 households from each PSU across the country [12]. The health facility survey was also conducted simultaneously and a sample size of 300 per type of facility was adequate to provide an estimate of 50% availability of essential medicines and technologies (assumed as no prior information was available) with a relative precision of 15% at 95% confidence level (CI). Thus, the health facilities serving the eligible population of the selected 600 PSUs were assessed for their preparedness to tackle NCDs.

The NNMS received ethical clearance from the Ethics review committee of the ICMR-NCDIR as well as from the respective institutional ethics committees of all the implementing agencies. The oversight of the survey was provided by an independent national technical working group, while the MoHFW, GoI defined the scope of the survey and provided funds.

The health facility preparedness was defined as the capacity of the facility to deliver services based on the availability of trained human resources, guidelines, essential medicines and technologies [13]. The survey covered four types of health facilities (three public namely PHC/ dispensary, CHC and DH) and one private primary in each of the 600 identified PSUs. A public facility meant a facility run by the government or supported by the government, and the rest were considered private. Only private facilities with an inpatient admission facility of 5–30 beds and those that did not provide specialized NCD care were included. Public facilities that served the selected PSU or those that were closest to it in urban and rural areas were included. If two PSUs fell in the same district, the shared facility was included under either of the PSUs based upon its accessibility. The study facilities were identified in consultation with the district health officials. A line listing of primary private health facilities within 3 miles of selected PSUs was prepared on the first day of the visit to the PSU along with PSU mapping and household listing process. From this list of private health facilities, one facility was selected in this order — within the PSU, closest to the PSU, closest to the PHC or closest to the CHC.

Prior permissions were sought from the state health government and communications were made with the district officials for facilitation of the survey. The research officer briefed about the survey to the medical officers of private and public facilities and sought their consent to participate. The health facility survey was carried out by the senior most member of the field team — a person with a Master’s in Social Work. All of them were trained in survey methodology and data collection procedures.

We used the global standard WHO-Service Availability and Readiness Assessment (SARA) tools for the health facility survey. They were adapted to suit the Indian context by expanding the number of conditions and items to reflect our requirements according to the National NCD Monitoring Framework and Action Plan [13, 14]. Separate study tools were used for primary public, secondary public and private primary facilities. The questionnaires included sections on basic infrastructure, provision of NCD related services, actual load of patients in a month, human resource availability and their training under the NPCDCS (only for public facilities), laboratory services, availability of medicines and technologies. The tools underwent face and content validity checks by experts in the national technical working group. A feasibility study was undertaken by the implementing institutions in selected facilities prior to the main survey. In the feasibility study, physical checks on availability were carried out to substantiate reporting of availability of medicines and technologies. To ensure the quality of information being collected, periodic supervisory visits were made by the local team, expert members of the core and technical working groups.

The list of essential medicines and technology for primary care facilities was defined according to the WHO-PEN recommendations [15]. In case of the secondary facilities we followed the definitions as per the NPCDCS guidelines [16]. Availability of any medicine or technology was considered if the pharmacist or the storekeeper reported it as being generally available within the premises of the facility either free of cost or on payment. Information on the availability of human resources and NCD services were collected from the Medical Officer/Office Clerk; medicines and technologies from the storekeeper or the pharmacist; and provision of laboratory services from the laboratory technician.

All survey data were collected by face-to-face interviews using Open Data Kit (ODK) technology on Android tablet devices and saved into comma-separated files (csv). Data analyses were conducted using IBM SPSS Version 22.0 (IBM Corp, Armonk, NY, USA). All the variables were summarized as proportions with 95% CIs and presented separately by location (rural/urban) and sector (public/private) for primary care facilities. Continuous variables which were normally distributed were presented as mean with 95% CI or as median (Inter Quartile Range - IQR). For secondary facilities where only public facilities were studied, we compared preparedness between facilities covered under NPCDCS with those not covered. This was based upon the information available with the NCD Division of the Ministry of Health and Family Welfare, Government of India.

## Results

**Fig. 1:**
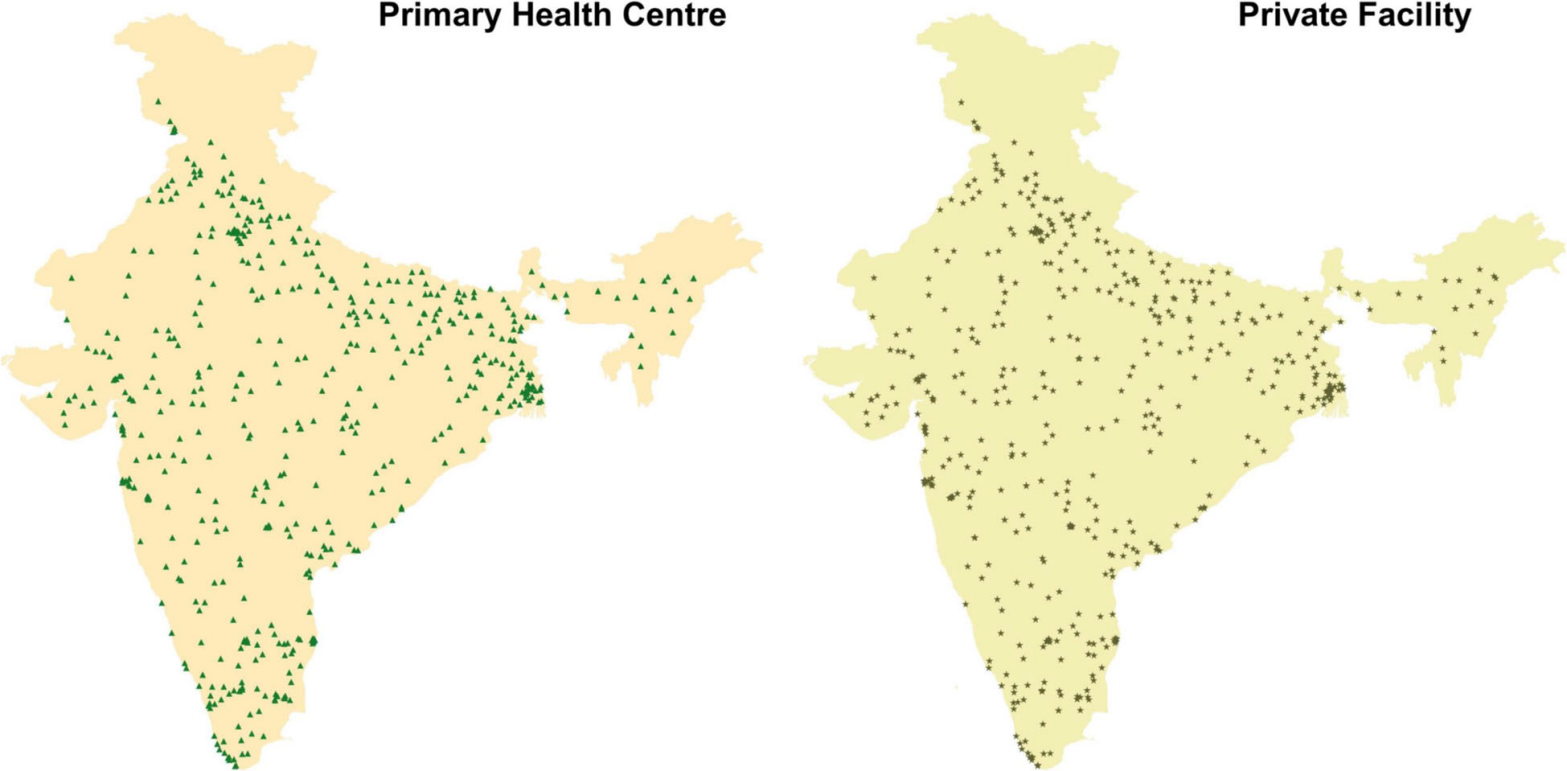
NNMS 2017-18: Surveyed public and private primary level health facilities

**Fig. 2:**
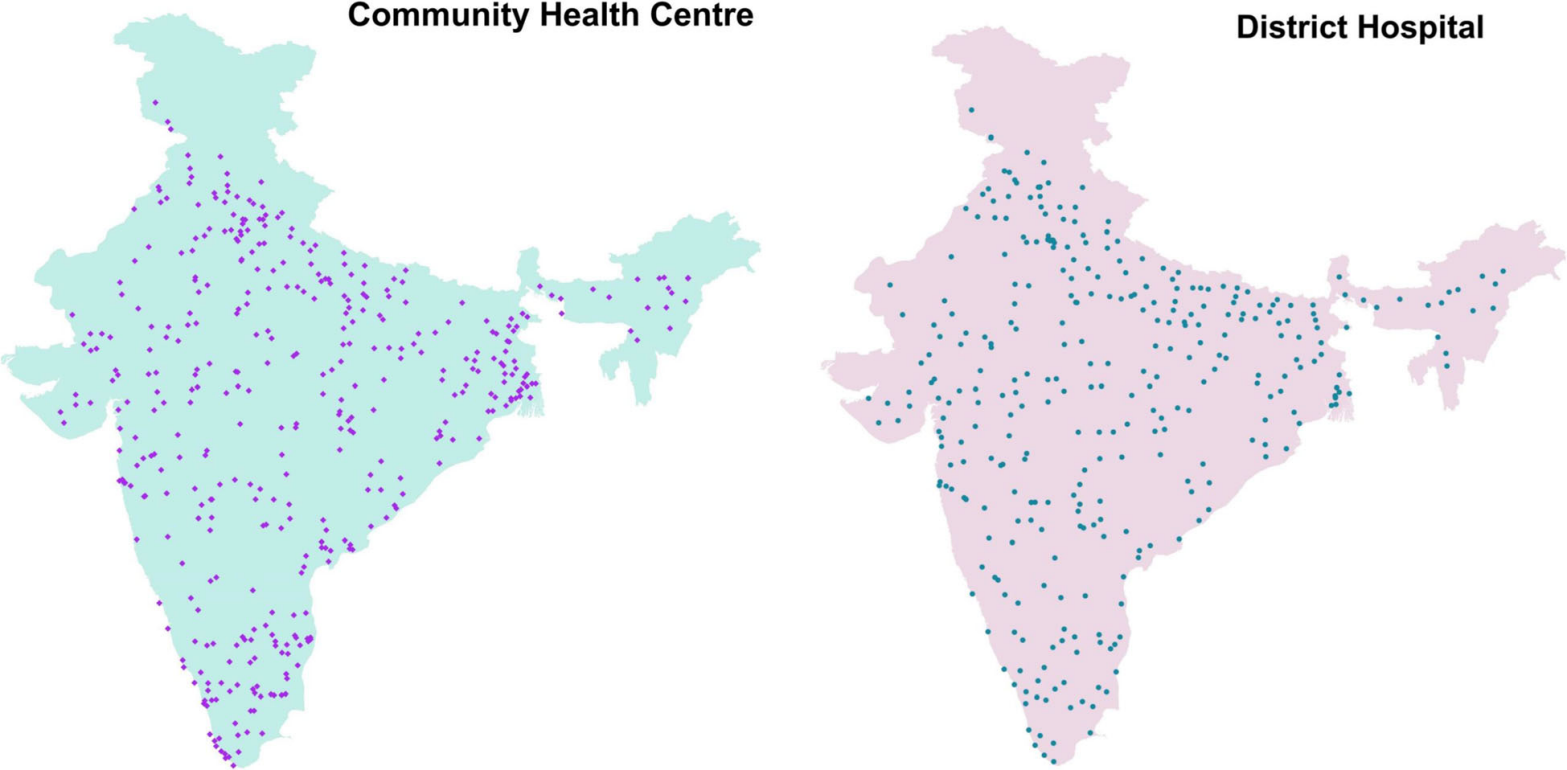
NNMS 2017-18: Surveyed public secondary level health facilities

A total of 537 primary public facilities (257 urban and 280 rural) and 527 private primary facilities (urban 277 and rural 235), 415 CHCs and 335 DHs were surveyed (Fig. 1) and (Fig. 2). All identified public facilities responded to the survey, while 15 private primary facilities refused, mainly due to concerns about divulging their data to outside agencies. In the rest of the PSUs, no private/public facility could be identified within approximately 3 miles radius. Only 512 primary level private facilities have been included for the final analyses.

### Essential medicines and technologies in primary (public and private) and secondary care facilities (public)

Overall, the availability of all major NCDs related to essential technologies and medicines for NCDs in primary care varied from 1.1% (95% CI; 0.3–3.3) in rural to 2.3% (95% CI; 1.0–5.1) in urban public facilities and 6.8% (95% CI; 4.2–10.8) in rural to 9.0% (95% CI; 6.2–13.0) in urban private facilities (Table 1). Private facilities fared much better in the availability of medicines (33.2–42.6%) as compared to public facilities (2.1–4.3%) and public facilities (38.1%) were better in technology availability than the private facilities (11.5–14.8%).

**Table 1:** Availability (%) of essential medicines and technologies for major NCDs at primary care facilities

Disease	Items & Definition	Public Primary			Private Primary		
		Urban ***N*** = 257	Rural ***N*** = 280	Total ***N*** = 537	Urban ***N*** = 277	Rural ***N*** = 235	Total ***N*** = 512
		Availability (95% CI)					
Diabetes mellitus	Medicine - Available medicines for Diabetes are Metformin and Insulin.	21.0 (16.4–26.4)	20.4 (16.0–25.5)	20.7 (17.4–24.3)	62.1 (56.2–67.6)	57.0 (50.6–63.2)	59.8 (55.4–63.9)
	Technologies - At least one of each technology related to Diabetes, including glucometer, glucostrips and urine strips, should be available at the facility.	47.5 (41.4–53.6)	52.9 (47.0–58.7)	50.3 (46.0–54.5)	87.4 (82.9–90.8)	79.6 (73.9–84.3)	83.8 (80.3–86.7)
	Both Medicines & Technologies as defined above	10.9 (7.6–15.3)	14.3 (10.6–18.9)	12.7 (10.1–15.8)	59.6 (53.7–65.2)	51.9 (45.5–58.3)	56.1 (51.7–60.3)
Cardio-vascular diseases	Medicine - Available medicines for Hypertension and CVDs are Aspirin, at least one ‘long acting calcium channel blocker, ACE inhibitor, or diuretic’ and at least one statin.	37.4 (31.6–43.5)	24.6 (19.9–30.0)	30.7 (27.0–34.8)	46.2 (40.4–52.1)	40.4 (34.3–46.8)	43.6 (39.3–47.9)
	Technologies - At least one of each technology related to Hypertension and CVDs, including BP apparatus, weighing scale and height scale, stethoscope, should be available at the facility.	67.7 (61.7–73.2)	60.0 (54.1–65.6)	63.7 (59.5–67.9)	14.8 (11.1–19.5)	12.3 (8.7–17.2)	13.7 (11.0–16.9)
	Both Medicines & Technologies as defined above	32.7 (27.2–38.7)	19.3 (15.1–24.3)	25.7 (22.2–29.6)	10.1 (7.1–14.3)	7.7 (4.9–11.8)	9.0 (6.8–11.8)
Chronic Respiratory Diseases	Medicine - Available medicines for COPD are at least one Bronchodilator and a steroid inhalant.	15.6 (11.6–20.5)	13.9 (10.3–18.5)	14.7 (12.0–18.0)	59.9 (54.0–65.6)	52.8 (46.4–59.1)	56.6 (52.3–60.9)
	Technologies - At least one ‘Stethoscope’, should be available at the facility for COPD.	99.2 (96.9–99.8)	99.3 (97.2–99.8)	99.3 (98.0–99.7)	90.3 (86.1–93.2)	87.2 (82.3–90.9)	88.9 (85.8–91.3)
	Both Medicines & Technologies as defined above	15.6 (11.6–20.5)	13.9 (10.3–18.5)	14.7 (12.0–18.0)	57.0 (51.1–62.8)	51.5 (45.1–57.8)	54.5 (50.1–58.8)
All above NCDs	Medicine - See above for disease-specific definitions	4.3 (2.4–7.6)	2.1 (1.0–4.7)	3.2 (2.0–5.0)	42.6 (36.9–48.5)	33.2 (27.4–39.5)	38.3 (34.2–42.6)
	Technologies - See above for disease-specific definitions	38.1 (32.4–44.2)	38.2 (32.7–44.1)	38.2 (34.1–42.4)	14.8 (11.1–19.5)	11.5 (8.0–16.3)	13.3 (10.6–16.5)
	Both Medicines & Technologies Both as defined above	2.3 (1.0–5.1)	1.1 (0.3–3.3)	1.7 (0.9–3.2)	9.0 (6.2–13.0)	6.8 (4.2–10.8)	8.0 (5.9–10.7)

The NPCDCS was being implemented in majority of the CHCs (72.8%) and DHs (86.8%). No major differences were noted on any of the indicators between districts where NPCDCS was being implemented versus those not implemented (Table 2).

**Table 2:** Availability (%) of essential medicines and technologies for NCDs at public secondary care facilities

Disease	Items based on NPCDCS guidelines	Community Health Centre			District Hospitals		
		NPCDCS^**a**^ (***n*** = 281)	Others (***n*** = 105)	Total ***N*** = 415	NPCDCS^**a**^ (***n*** = 290)	Others (***n*** = 45)	Total ***N*** = 335
			Availability (95% CI)				
Diabetes mellitus	Medicines - At least one of each - hypoglycemic agent and insulin	55.2 (49.3–60.9)	43.3 (35.1–51.8)	51.3 (46.5–56.1)	74.5 (69.1–79.2)	73.3 (58.6–84.2)	74.3 (69.4–78.7)
	Technologies - At least one of each - lancets. Glucometer, biochemical analyser, glucostrips, urine strips reagents/kits for glucose test and for lipid profile, centrifuge	21.7 (17.3–26.9)	13.4 (8.6–20.3)	19.0 (15.5–23.1)	50.3 (44.6–56.1)	51.1 (36.8–65.3)	50.4 (45.1–55.8)
	Both medicines & technologies as defined above	17.1 (13.1–22.0)	9.0 (5.1–15.1)	14.5 (11.4–18.2)	42.1 (36.5–47.8)	42.2 (28.8–56.9)	42.1 (36.9–47.5)
Cardio-vascular diseases	Medicines - At least one each - ‘anti platelet agent, betablocker, long acting calcium channel blocker, ACE inhibitor, diuretic, nitrate, statin / lipid lowering medicines, medicines for shock and heart failure.	39.9 (34.3–45.7)	21.6 (15.5–29.4)	34.0 (29.6–38.7)	59.0 (53.2–64.5)	62.2 (47.4–75.1)	59.4 (54.0–64.5)
	Technologies - At least one of each - B.P. Apparatus, Stethoscope, Measuring Tape, weighing scale, Height scale, Cardiac Monitor, Defibrillator, ECG machine & Roll, 12 Channel stress ECG Tread Mill.	1.1 (0.3–3.3)	2.2 (0.7–6.7)	1.4 (0.6–3.2)	20.3 (16.1–25.4)	11.1 (4.7–24.1)	19.1 (15.2–23.7)
	Both medicines & technologies as defined above	1.1 (0.3–3.3)	0.7 (0.1–5.1)	1.0 (0.4–2.5)	16.6 (12.7–21.3)	8.9 (3.4–21.4)	15.5 (12.0–19.8)
Chronic Respiratory Diseases	Medicines – At least one of each - Bronchodilator, a steroid inhalant.	19.2 (15.0–24.3)	17.9 (12.3–25.4)	18.8 (15.3–22.9)	36.6 (31.2–42.3)	31.1 (19.3–45.9)	35.8 (30.9–41.1)
	Technologies - At least one of each - Nebulizer and Pulse Oximeter.	75.4 (70.1–80.1)	61.9 (53.4–69.8)	71.1 (66.5–75.3)	94.5 (91.2–96.6)	84.4 (70.8–92.4)	93.1 (89.9–95.4)
	Both medicines & technologies as defined above	17.4 (13.4–22.3)	15.7 (10.4–22.9)	16.9 (13.6–20.8)	35.2 (29.9–40.9)	31.1 (19.3–45.9)	34.6 (29.7–39.9)
All above NCDs	Medicines - Inclusive of above medicines for specific diseases	13.2 (9.7–17.7)	8.2 (4.6–14.2)	11.6 (8.8–15.0)	28.3 (23.4–33.7)	22.2 (12.4–36.6)	27.5 (22.9–32.5)
	Technologies - Inclusive of above technologies for specific diseases	0.4 (0.0–2.5)	0.7 (0.1–5.1)	0.5 (0.1–1.9)	14.5 (10.9–19.0)	6.7 (2.2–18.8)	13.4 (10.2–17.5)
	Both medicines & technologies as defined above	0.4 (0.0–2.5)	0.7 (0.1–5.1)	0.5 (0.1–1.9)	14.5 (10.9–19.0)	6.7 (2.2–18.8)	13.4 (10.2–17.5)

^a^Facilities where National Program for Prevention and Control of Cancer, Diabetes and Cardio-vascular Diseases including Stroke has been officially implemented and others are where it has been not officially implemented and status unknown

For diabetes, essential medicines were available in one-fifth of the primary public facilities, three-fifths of private ones, three-fourth of district hospitals and about half of CHCs (Tables 1 and 2). Metformin was available in 77.1% of public facilities, 91% of CHCs and DHs. Insulin was available in only 21.4% of PHCs, most DHs (82.7%) and about half (56.4%) of CHCs (Additional Table 1). The availability of essential technology in both private and public facilities was higher than the availability of medicines. Only 10.9–14.3% of urban and rural public primary facilities had both medicine and technologies (Table 1). About half of the DHs had all essential technologies for diabetes while this was in less than 22% of CHCs. NPCDCS implementing CHCs had a 6% higher availability of both medicines and technologies for diabetes than non-implementing CHCs (Table 2).

For CVDs, the proportion of public primary facilities with essential technologies (63.7%) was two times higher than essential medicines (30.7%). The urban public primary facilities had a 13% higher availability of both medicines and technologies (Table 1). Among the secondary public facilities, almost all CHCs were inadequate to manage CVDs, but essential medicines were available in 59% and 39.9% of NPCDCS implementing DHs and CHCs, respectively. Amongst the NPCDCS implementing and non-implementing secondary public facilities, nearly 20% difference in availability of medicines in CHCs and 9% difference in availability of technologies for DHs was observed (Table 2). Amlodipine and atenolol were the most available drugs for CVDs at PHCs, CHCs and DHs. Adult weighing scale, blood pressure apparatus and stethoscope were universally available in PHCs. Twelve Channel stress ECG treadmill machine was available only in a third of the district hospitals (30.4%), while other equipment were generally available (Additional Tables 1 and 2).

CRD fared the best in available technologies than medicines as only a stethoscope was required as per the guidelines for PHCs, pulse oximeter and nebulizer was near-universal in CHCs and DHs (Tables 1 and 2). NPCDCS districts were 10–13% better prepared in technology availability than non-implementing others with minimal differences in availability of medicines for CRDs (Table 2). Salbutamol tablet was available in more than 80% of public primary and secondary care facilities and theophylline + etophylline combination (Deriphyllin) in 70% of PHCs and more than 80% of CHCs and DHs. Salbutamol inhaler was available in 54.5% CHCs and 33.7% of the PHCs and only half of among these had steroid inhaler available (CHCs: 27.7% and PHCs: 15.1%) (Additional Table 1).

Almost none of the CHCs were equipped to manage all the three major NCDs largely due to deficiencies in technologies, though medicine availability was also poor. 14.5% (95% CI;10.9–19.0) of the DHs where NPCDCS was being implemented and 6.8% (95% CI; 2.2–19.2) of other DHs were fully equipped to manage all the three major NCDs (Table 2).

### Availability of human resources in public primary and secondary care facilities

Nearly 15% of PHCs (similar among urban and rural), 8% CHCs and 7% DHs had no MBBS duty doctor and slightly more than 50% public secondary care facilities and 29.2% of PHCs (higher proportion in rural: 35.0%) had a doctor from alternative systems of medicine (Table 3 and Additional Table 3). Availability of nurse-midwife or equivalent was higher in rural (rural: 76.4% and urban: 70.0%) PHCs, staff nurse was highest in urban PHCs (urban: 73.5% and rural: 67.5%) (Additional Table 3) The proportion of staff available at CHCs was similar to district hospitals, except for specialists. Most trained were the medical officers (30.4%) in PHCs and staff nurses (23.9%) in both CHCs and DHs (20.6%) (Table 3).

**Table 3:** Availability (%) of technical human resources in public health facilities in India NNMS (2017–18)

Staff Category	PHC (***n*** = 537)		CHC (*n* = 415)		DH (*n* = 335)	
	% (95% CI) of Health facility where mentioned staff is					
Doctors	Available	Trained	Available	Trained	Available	Trained
**General Duty Medical Officers**						
Allopathic System	85.1 (81.8–87.9)	30.4 (26.6–34.4)	91.6 (88.5–93.9)	21.0 (17.3–25.2)	93.4 (90.2–95.6)	15.8 (12.3–20.1)
AYUSH* system	29.2 (25.5–33.2)	5.2 (3.6–7.5)	55.9 (51.1–60.6)	7.5 (5.3–10.4)	53.4 (48.1–58.7)	6.6 (4.4–9.8)
**Specialist Medical Officers**						
Medicine	10.8 (7.1–18.3)	3.4 (1.9–6.3)	25.1 (21.1–29.5)	4.6 (2.9–7.1)	79.4 (74.7–83.4)	16.4 (12.8–20.8)
Surgery	3.2 (1.8–6.1)	0.0	27.2 (23.2–31.7)	3.6 (2.2–5.9)	88.4 (84.5–91.4)	11.3 (8.4–15.2)
Obs & Gynae	12.3 (9.2–17.6)	1.9 (1.0–3.4)	44.6 (39.8–49.4)	7.0 (4.9–9.9)	81.8 (77.3–85.6)	13.7 (10.4–17.9)
Ophthalmologist	8.0 (5.5–13.9)	1.3 (0.6–2.7)	21.4 (17.8–25.7)	2.4 (1.3–4.4)	81.5 (77.0–85.3)	9.6 (6.8–13.2)
**Nursing and paramedical Staff**						
Staff Nurse	70.4 (66.4–74.1)	23.5 (20.1–27.2)	97.8 (95.9–98.9)	23.9 (20.0–28.2)	97.0 (94.5–98.4)	20.6 (16.6–25.3)
Health Assistants or equivalent	46.2 (42.0–50.4)	11.0 (8.6–13.9)	51.8 (47.0–56.6)	9.2 (6.7–12.3)	40.6 (35.5–46.0)	5.4 (3.4–8.4)
Auxiliary Nurse Midwife or equivalent	73.4 (69.5–76.9)	16.6 (13.7–20.0)	76.6 (72.3–80.5)	13.5 (10.5–17.1)	63.9 (58.6–68.9)	9.6 (6.8–13.2)
Pharmacist	81.9 (78.4–85.0)	14.5 (11.8–17.8)	94.9 (92.4–96.7)	11.8 (9.0–15.3)	99.1 (97.3–99.7)	9.9 (7.1–13.5)
Lab Technician	70.8 (66.8–74.5)	14.3 (11.6–17.6)	94.5 (91.8–96.3)	11.8 (9.0–15.3)	98.8 (96.9–99.6)	13.1 (9.9–17.2)
Physiotherapist	1.5 (0.7–3.0)	0.0	20.5 (16.9–24.6)	2.2 (1.1–4.1)	75.5 (70.6–79.8)	10.7 (7.8–14.5)
Care coordinator	3.2 (2.0–5.0)	0.9 (0.4–2.2)	5.8 (3.9–8.5)	2.2 (1.1–4.1)	11.0 (8.1–14.9)	1.5 (0.6–3.5)
Counsellor	8.6 (6.5–11.3)	2.6 (1.5–4.4)	56.1 (51.3–60.9)	9.4 (6.9–12.6)	80.9 (76.3–84.8)	13.7 (10.4–17.9)

*Alternative systems of medicine - Ayurveda, Yoga and Naturopathy, Unani, Siddha and Homeopathy

### NCD services in public primary and secondary care facilities

Most district hospitals provided ambulatory (100.0%), in-patient (96.7%) and emergency services (93.7%), as were the majority of CHCs (Table 4). Rural PHCs were better prepared with these services than the urban PHCs (Additional Table 4) In terms of the range of services, 87.9% of PHCs, 92.0% of CHCs and 98.8% of DHs provided screening services for NCDs. We observed nearly half the percentage reduction in counselling services offered at DHs (62.1%) to CHCs (32.8%) and then to PHCs (15.3%). Physiotherapy services were available at more than 75% of DHs and only in 21.2% CHCs. Laboratory services for major NCDs were near-universal among both public secondary facilities (CHCs: 94.0% and DHs: 99.7%), while only 69.3% public primary facilities provided them. On average, PHCs were registering 83 new NCD patients per month in their outpatients (urban - 103 and rural – 61 patients), compared to 275 at CHCs and 756 at DHs. Only 8 patients were being admitted for NCDs per month in CHCs and 75 patients in DHs (Table 4).

**Table 4:** Noncommunicable diseases (NCD) related services being provided in the study facilities

NCD Services	Public Primary	Public Secondary	
		CHCs	DHs
	*N* = 537	*N* = 415	*N* = 335
	% (95% CI)		
Ambulatory care	70.8 (66.8–74.5)	94.2 (91.5–96.2)	100.0 (100.0–100.0)
In-patient care	52.0 (47.7–56.2)	92.3 (89.3–94.5)	96.7 (94.2–98.2)
Emergency care	40.6 (36.5–44.8)	82.2 (78.2–85.6)	93.7 (90.6–95.9)
Screening for NCDs	87.9 (84.8–90.4)	92.0 (89.0–94.3)	98.8 (96.9–99.6)
Counselling for NCDs	15.3 (12.5–18.6)	32.8 (28.4–37.4)	62.1 (56.8–67.1)
Physiotherapy	3.7 (2.4–5.7)	21.2 (17.5–25.4)	75.2 (70.3–79.6)
Laboratory testing for major NCDs	69.3 (65.2–73.0)	94.0 (91.2–95.9)	99.7 (97.9–100.0)
Availability of management guidelines in the hospital	37.1 (33.1–41.2)	46.5 (41.7–51.3)	64.8 (59.5–69.7)
Display of NCD related IEC materials inside the hospital	62.9 (58.8–66.9)	77.1 (72.8–80.9)	84.8 (80.5–88.2)
**Median (IQR) NCD patient load per month**			
New Out-patients	83 (252)	275 (696)	756 (1536)
NCD admissions	0 (9)	8 (37)	75 (241)

## Discussion

This is the first nationally representative health facility survey that assessed the preparedness of public and private primary and public secondary care facilities in India to address NCDs as part of the global and national NCD monitoring framework. While disease specific preparedness, especially for diabetes and CVDs is modest, a comprehensive package of medicines, technologies and human resources to deliver services for the three major NCDs (excluding cancer) still eludes India.

We defined the availability as always and generally available in the facility as reported by the pharmacist, while a definition that is often used is ‘available on the day of the visit’. There is a need for standardization in medicine availability survey methodologies [17]. CRD management guidelines as a part of the NPCDCS or WHO-PEN Package were not available at the time of the survey. They have been developed since then (2018) and include the use of peak flowmeter or spirometer for their diagnosis along with nebulizers and pulse oximeters for their management and monitoring at primary/secondary facilities [18]. We could not derive the consolidated indicator for proportion of primary care facilities which had essential medicines and technologies as there is no listing of private primary care facilities in India.

A study using the WHO-SARA methodology in Bangladesh, Haiti, Malawi, Nepal, and Tanzania in 2013–15 reported that very few facilities were fully “ready” to provide any one NCD service due to critical shortages of trained health workers and essential medicines [19]. Similar deficiencies in human resources and essential medicines and technologies have also been reported from other developing countries [20–23]. Similar to our findings on poor availability of medicines for CRD, global estimates on the availability of medicines for asthma was 30.1% and 43.1% in the public and private sectors respectively [24].

Earlier studies in NPCDCS have highlighted sub-optimal training or competencies regarding NPCDCS, shortage of necessary medicines and equipments [25–29]. Many countries in the South-east region have implemented the WHO-PEN with varying degrees of success and have identified barriers in its implementation that include availability of essential technologies and medicines [30–32].

Human resources play a major role in making the services available in health facilities. In India at primary health care facilities, only a medical graduate is expected to work. Our study shows that some PHCs reported availability of specialists also. These must be upgraded PHCs or even CHCs serving additionally as a PHC or it also indicates inappropriate posting policy of the doctors not commensurate with their training that has been documented earlier [33]. Additionally, human resource allocations are usually influenced by health policies and disease epidemiology at sub-national and local levels. Furthermore, the nature of appointment could be either visiting or contractual [34, 35].

We found no major differences in the public facilities where NPCDCS was implemented versus those without and those not under it. Also, several states especially those of southern India, are in advanced epidemiological transition and have effectively begun several state level initiatives to address NCDs. We further analyzed data to look into differences between NPCDCS implementing districts and not to evaluate NPCDCS. Furthermore, the gaps in health facility readiness in terms of medicine and equipment availability and trained manpower has already been commented upon by other researchers [36].

Public primary care facilities were more equipped in terms of essential technologies, while the public secondary care facilities are better equipped in availability of essential medicines for the three major NCDs — DM, CVDs, and CRD. Amongst both primary and secondary public care facilities, the district hospitals were better prepared with essential technologies and medicines for diabetes mellitus. Screening and laboratory services for NCDs are being provided by a large percentage of PHCs, CHCs and DHs demonstrating programmatic initiatives by the Government of India to prevent and control NCDs. India is still preparing to address the three major NCDs (DM, CVD and CRD), cancer and other NCDs – especially mental health disorders and chronic kidney diseases will require immediate attention. India, after initial exclusion of primary care facilities in its approach to managing NCDs, has done a course correction and has now included NCDs as the entry point for its comprehensive primary health care strategy to achieve universal health coverage. The strategy includes revamping the sub-centres and PHCs into Health and Wellness Centres (HWCs) with a specific focus on additional human resources and essential medicines and technologies [37]. Necessary flexibilities in workforce recruitment and retaining strategies along with the use of blended models of self-learning using virtual training courses on Massive Open Online Courses (MOOC) platforms are being introduced under the National Health Mission [38].

The use of a comprehensive and global standard tool by trained field staff visiting the facility; support from MoHFW, GoI to undertake the health facility assessment; collaboration and coordination from all the state and district health officials were the strengths of this study.

The key limitation was use of reported data that was not verified physically. Others include, not covering other health facility components on finances, revenues, quality of care or patient interviews. As the survey was based upon the National NCD Monitoring Framework and Action Plan, we covered important components of health facility surveys in terms of infrastructure, services, equipment and human resources. There is scope to include private sector, tertiary care hospitals, other NCDs, also expand the data collection tools to cover additional health system indicators and definitions of availability in future surveys.

With this survey, we have established a baseline to evaluate future developments in health systems for NCDs through repeated surveys. Health facility surveys for NCD preparedness as standalone are not cost-effective. They need to be integrated into NCD risk factor surveys (as was NNMS) or with a general health facility preparedness survey. In the Indian context, given the verticality of many disease-specific national initiatives, the current model has a better chance of success, which would apply to other developing countries in similar situations. Such surveys are useful in monitoring the progress being made in the preparedness of the health facilities as well as in evaluating the effectiveness of the national programs.

## Conclusion

In conclusion, this survey has documented significant gaps and strengths of the health system response to NCDs in India. The gaps in terms of availability of essential medicines, technologies, training of available human resources and counselling services for NCDs. Documented strengths are the better performance of facilities in availability of screening and laboratory services for NCDs. This survey provides the baseline against which all future assessments will be used to measure progress towards universal health coverage for NCDs. Appropriate resource allocation can be guided by the results. Furthermore, it provides health authorities plan for setting up surveillance systems for NCDs. This study will find relevance in many similar countries like India and would give an impetus to improve NCD prevention and control efforts.

## Supplementary Information

**Additional file 1: Additional Table 1**. Proportion of public health care facilities where specific medicines were always or generally available.

**Additional file 2: Additional Table 2**. Proportion of public health care facilities where specific technologies were always or generally available.

**Additional file 3: Additional Table 3**. Availability (%) of technical human resources in public primary urban and rural health facilities in India; NNMS (2017–18).

**Additional file 4: Additional Table 4**. Noncommunicable diseases (NCD) related services being provided in the public primary urban and rural study facilities; NNMS (2017–18).

## Data Availability

The National Noncommunicable Disease Monitoring Survey (NNMS) report is available at https://www.ncdirindia.org/nnms/. Further data are available upon request.

## Abbreviations

NNMS: National Noncommunicable Disease Monitoring Survey
NCDs: Noncommunicable Diseases
NPCDCS: National Program for Prevention and Control of Cancer, Diabetes, Cardio-vascular diseases and Stroke
WHO: World Health Organization
SARA: Service availability and readiness assessment
PHC: Primary health care
CHC: Community health centre
DH: District hospital
PSUs: Primary Sampling Units
ODK: Open Data Kit
ICMR: Indian Council of Medical Research
MOOC: Massive Open Online Courses
NCDIR: National Centre for Disease Informatics and Research
GoI: Government of India
DM: Diabetes Mellitus
CVD: Cardiovascular Diseases
CRD: Chronic Respiratory Diseases
