# Supplementary material for: Preparedness of primary and secondary health facilities in India to address major noncommunicable diseases: results of a National Noncommunicable Disease Monitoring Survey (NNMS)
Source: BMC Health Serv Res. 2021 Jul 31;21:757. doi: 10.1186/s12913-021-06530-0 (PMC8325187; doi:10.1186/s12913-021-06530-0)
Supplement: Supplementary file 1 — Additional file 1: Additional Table 1. Proportion of public health care facilities where specific medicines were always or generally available. [file 12913_2021_6530_MOESM1_ESM.docx]

Additional Table 1. Proportion of public health care facilities where specific medicines were always or generally available

| **Medicines** | **Primary public health care facilities** | | | **Secondary public** **health care facilities** | |
| --- | --- | --- | --- | --- | --- |
|  | **Urban**  **N = 257** | **Rural**  **N = 280** | **Total**  **N = 537** | **CHC**  **N = 415** | **DH**  **N = 335** |
|  | **% (95% CI)** | | | | |
| **Cardio-vascular Diseases** | | | | | |
| Tab Aspirin (low dose) | 49.8  (43.7-55.9) | 47.9  (42.0-53.7) | 48.8  (44.6-53.0) | 66.3  (61.6-70.7) | 76.7  (71.9-80.9) |
| Tab Clopidogrel | 26.8  (21.8-32.6) | 15.4  (11.6-20.1) | 20.9  (17.6-24.5) | 41.9  (37.3-46.7) | 67.8  (62.6-72.6) |
| Tab. Atenolol | 74.3  (68.6-79.3) | 77.9  (72.6-82.4) | 76.2  (72.4-79.6) | 83.6  (79.7-86.9) | 93.1  (89.9-95.4) |
| Tab. Metoprolol | 29.2  (23.9-35.1) | 29.6  (24.6-35.3) | 29.4  (25.7-33.4) | 46.3  (41.5-51.1) | 63.9  (58.6-68.9) |
| Tab. Amlodipine | 81.7  (76.5-86.0) | 80.4  (75.3-84.6) | 81.0  (77.5-84.1) | 92.8  (89.8-94.9) | 93.4  (90.2-95.6) |
| Tab. Verapamil | 9.3  (6.3-13.6) | 6.1  (3.8-9.6) | 7.6  (5.7-10.2) | 18.3  (14.9-22.3) | 31.6  (26.9-36.8) |
| Tab. Captopril | 7.0  (4.5-10.9) | 4.3  (2.4-7.4) | 5.6  (3.9-7.9) | 10.1  (7.6-13.4) | 16.1  (12.6-20.5) |
| Tab. Enalapril | 40.9  (35.0-47.0) | 29.6  (24.6-35.3) | 35.0  (31.1-39.2) | 51.6  (46.7-56.4) | 68.4  (63.2-73.1) |
| Tab. Isosorbide Dinitrate | 41.2  (35.4-47.4) | 36.4  (31.0-42.2) | 38.7  (34.7-42.9) | 64.3  (59.6-68.8) | 87.5  (83.5-90.6) |
| Glyceryl Trinitrate, Sub lingual tabs | 10.5  (7.3-14.9) | 10.4  (7.3-14.5) | 10.4  (8.1-13.3) | 20.7  (17.1-24.9) | 41.5  (36.3-46.9) |
| Tab. Atorvastatin / Any statin | 49.0  (42.9-55.1) | 30.7  (25.6-36.4) | 39.5  (35.4-43.7) | 59.3  (54.5-63.9) | 80.9  (76.3-84.8) |
| Tab. Frusemide | 57.2  (51.1-63.1) | 67.5  (61.8-72.7) | 62.6  (58.4-66.6) | 77.1  (72.8-80.9) | 88.7  (84.8-91.6) |
| Chlorothiazide / Thiazide | 21.0  (16.4-26.4) | 15.4  (11.6-20.1) | 18.1  (15.0-21.6) | 31.3  (27.0-36.0) | 38.2  (33.1-43.5) |
| Spironolactone | 9.3  (6.3-13.6) | 9.3  (6.4-13.3) | 9.3  (7.1-12.1) | 24.8  (20.9-29.2) | 44.5  (39.2-49.9) |
| Tablet Digoxin | 19.8  (15.4-25.2) | 22.9  (18.3-28.2) | 21.4  (18.1-25.1) | 42.9  (38.2-47.7) | 69.0  (63.8-73.7) |
| Tab. Methyldopa | 26.1  (21.1-31.8) | 32.9  (27.6-38.6) | 29.6  (25.9-33.6) | 54.0  (49.1-58.7) | 65.7  (60.4-70.6) |
| **Diabetes Mellitus** | | | | | |
| Tab. Glibenclamide | 39.3  (33.5-45.4) | 41.8  (36.1-47.7) | 40.6  (36.5-44.8) | 54.0  (49.1-58.7) | 63.0  (57.7-68.0) |
| Tab. Glimepiride | 61.1  (55.0-66.9) | 49.3  (43.4-55.1) | 54.9  (50.7-59.1) | 70.1  (65.5-74.3) | 80.9  (76.3-84.8) |
| Insulin Regular /Intermediate | 22.2  (17.5-27.7) | 20.7  (16.4-25.9) | 21.4  (18.1-25.1) | 56.4  (51.6-61.1) | 82.7  (78.2-86.4) |
| Tab. Metformin | 79.0  (73.6-83.6) | 75.4  (70.0-80.1) | 77.1  (73.3-80.5) | 91.3  (88.2-93.7) | 91.0  (87.5-93.7) |
| **Chronic Respiratory Diseases** | | | | | |
| Tab. Deriphyllin | 68.1  (62.1-73.5) | 71.8  (66.2-76.8) | 70.0  (66.0-73.8) | 81.0  (76.9-84.5) | 81.8  (77.3-85.6) |
| Tab Prednisolone | 52.9  (46.8-59.0) | 61.1  (55.2-66.6) | 57.2  (52.9-61.3) | 73.0  (68.5-77.1) | 80.3  (75.7-84.2) |
| Tab. Salbutamol | 81.3  (76.1-85.6) | 87.1  (82.7-90.6) | 84.4  (81.0-87.2) | 86.0  (82.3-89.0) | 86.3  (82.1-89.6) |
| Inhaler Salbutamol | 33.5  (27.9-39.5) | 33.9  (28.6-39.7) | 33.7  (29.8-37.8) | 54.5  (49.6-59.2) | 69.3  (64.1-74.0) |
| Any Steroid Inhaler | 16.0  (12.0-21.0) | 14.3  (10.6-18.9) | 15.1  (12.3-18.4) | 27.7  (23.6-32.2) | 42.4  (37.2-47.8) |
| **Antibiotics** | | | | | |
| Inj. Crystalline / Benzathine penicillin | 22.2  (17.5-27.7) | 20.7  (16.4-25.9) | 15.5  (12.6-18.8) | 37.6  (33.0-42.4) | 42.1  (36.9-47.5) |
| Cap. Amoxicillin | 94.6  (91.0-96.8) | 91.1  (87.1-93.9) | 92.7  (90.2-94.7) | 95.2  (92.6-96.9) | 90.4  (86.8-93.2) |
| Tab. Azithromycin | 84.0  (79.0-88.0) | 80.0  (74.9-84.3) | 81.9  (78.4-85.0) | 89.6  (86.3-92.2) | 88.4  (84.5-91.4) |
| Tab.Erythromycin | 53.3  (47.2-59.3) | 52.9  (47.0-58.7) | 53.1  (48.8-57.3) | 61.9  (57.1-66.5) | 60.9  (55.6-66.0) |
| **Miscellaneous including emergency medicines** | | | | | |
| Tab. Folic Acid | 95.7  (92.4-97.6) | 92.1  (88.3-94.8) | 93.9  (91.5-95.6) | 93.5  (90.7-95.5) | 94.3  (91.3-96.4) |
| Alprazolam | 51.0  (44.9-57.1) | 52.9  (47.0-58.7) | 52.0  (47.7-56.2) | 66.7  (62.1-71.1) | 81.5  (77.0-85.3) |
| Tab. Diazepam | 40.9  (35.0-47.0) | 61.4  (55.6-67.0) | 51.6  (47.3-55.8) | 70.8  (66.3-75.0) | 77.6  (72.8-81.8) |
| Inj. Adrenaline | 58.0  (51.8-63.9) | 67.1  (61.4-72.4) | 62.8  (58.6-66.8) | 85.8  (82.1-88.8) | 91.3  (87.8-93.9) |
| Inj Aminophylline | 33.9  (28.3-39.9) | 43.2  (37.5-49.1) | 38.7  (34.7-42.9) | 58.8  (54.0-63.4) | 80.9  (76.3-84.8) |
| Inj Atropine sulphate | 51.0  (44.9-57.1) | 68.6  (62.9-73.8) | 60.1  (55.9-64.2) | 89.9  (86.6-92.4) | 91.3  (87.8-93.9) |
| Inj. Dexamethasone 2mg/ml vial | 68.9  (62.9-74.3) | 86.1  (81.5-89.7) | 77.8  (74.1-81.2) | 94.0  (91.2-95.9) | 94.6  (91.6-96.6) |
| Inj. Digoxin | 9.7  (6.6-14.0) | 10.7  (7.6-14.9) | 10.2  (7.9-13.1) | 22.4  (18.6-26.7) | 47.8  (42.4-53.1) |
| Inj. Glyceryl Trinitrate | 6.2  (3.8-9.9) | 4.6  (2.7-7.8) | 5.4  (3.8-7.7) | 17.8  (14.4-21.8) | 46.9  (41.6-52.2) |
| Inj. Heparin Sodium | 7.4  (4.8-11.3) | 5.4  (3.2-8.7) | 6.3  (4.6-8.7) | 21.4  (17.8-25.7) | 64.2  (58.9-69.1) |
| Inj. Mephenterine | 7.4  (4.8-11.3) | 12.9  (9.4-17.3) | 10.2  (7.9-13.1) | 32.8  (28.4-37.4) | 68.4  (63.2-73.1) |
| Inj. Streptokinase | 5.8  (3.5-9.5) | 4.3  (2.4-7.4) | 5.0  (3.5-7.2) | 21.9  (18.2-26.2) | 62.4  (57.1-67.4) |
| Inj. 20% Mannitol | 15.2  (11.3-20.1) | 33.6  (28.3-39.3) | 24.8  (21.3-28.6) | 66.0  (61.3-70.4) | 89.6  (85.8-92.4) |
| Inj. Lignocaine hydrochloride | 40.9  (35.0-47.0) | 72.5  (67.0-77.4) | 57.4  (53.1-61.5) | 89.6  (86.3-92.2) | 91.9  (88.5-94.4) |
| Inj. Diazepam | 40.5  (34.6-46.6) | 68.6  (62.9-73.8) | 55.1  (50.9-59.3) | 90.4  (87.1-92.9) | 93.4  (90.2-95.6) |
