# Supplementary material for: Preparedness of primary and secondary health facilities in India to address major noncommunicable diseases: results of a National Noncommunicable Disease Monitoring Survey (NNMS)
Source: BMC Health Serv Res. 2021 Jul 31;21:757. doi: 10.1186/s12913-021-06530-0 (PMC8325187; doi:10.1186/s12913-021-06530-0)
Supplement: Supplementary file 2 — Additional file 2: Additional Table 2. Proportion of public health care facilities where specific technologies were always or generally available. [file 12913_2021_6530_MOESM2_ESM.docx]

Additional Table 2. Proportion of public health care facilities where specific technologies were always or generally available

| **Technologies** | **Primary public health care facilities** | | | **Secondary public** **health care facility** | |
| --- | --- | --- | --- | --- | --- |
|  | **Urban**  **N = 257** | **Rural**  **N = 280** | **Total**  **N = 537** | **CHC**  **N = 415** | **DH**  **N = 335** |
|  | **% (95% CI)** | | | | |
| **Cardio-vascular diseases** | | | | | |
| Adult Weighing Scale | 98.8  (96.4-99.6) | 97.5  (94.8-98.8) | 98.1  (96.6-99.0) | 99.0  (97.5-99.6) | 99.1  (97.3-99.7) |
| Stadiometer | 67.7  (61.7-73.2) | 61.8  (55.9-67.3) | 64.6  (60.5-68.6) | 77.3  (73.1-81.1) | 88.1  (84.1-91.1) |
| Measuring Tape | 67.7  (61.7-73.2) | 76.4  (71.1-81.0) | 72.3  (68.3-75.9) | 80.5  (76.4-84.0) | 84.2  (79.9-87.7) |
| Blood Pressure measuring device | 99.6  (97.3-99.9) | 99.6  (97.5-100.0) | 99.6  (98.5-99.9) | 99.8  (98.3-100.0) | 100.0  (100.0-100.0) |
| Cardiac Monitor | 2.7  (1.3-5.6) | 3.6  (1.9-6.5) | 3.2  (2.0-5.0) | 33.7  (29.3-38.4) | 81.2  (76.6-85.0) |
| Defibrillator | 2.7  (1.3-5.6) | 3.9  (2.2-7.0) | 3.4  (2.1-5.3) | 25.1  (21.1-29.5) | 72.8  (67.8-77.3) |
| Electrocardiograph (ECG) machine | 16.3  (12.3-21.4) | 11.1  (7.9-15.3) | 13.6  (10.9-16.8) | 68.2  (63.5-72.5) | 96.7  (94.2-98.2) |
| 12 Channel stress ECG Tread Mill | NA | NA | NA | 7.5  (5.3-10.4) | 30.4  (25.7-35.6) |
| ECG Roll | 14.8  (10.9-19.7) | 8.2  (5.5-12.1) | 11.4  (8.9-14.3) | 47.2  (42.5-52.1) | 86.3  (82.1-89.6) |
| Reagents /kits for Lipid profile | 16.0  (12.0-21.0) | 8.9  (6.1-12.9) | 12.3  (9.8-15.4) | 37.6  (33.0-42.4) | 68.7  (63.5-73.4) |
| **Chronic Respiratory Diseases** | | | | | |
| Nebulizer | 51.4  (45.2-57.4) | 70.0  (64.4-75.1) | 61.1  (56.9-65.1) | 91.3  (88.2-93.7) | 98.5  (96.5-99.4) |
| Oxygen mask | 53.7  (47.6-59.7) | 77.5  (72.2-82.0) | 66.1  (62.0-70.0) | 97.6  (95.6-98.7) | 97.9  (95.7-99.0) |
| Oxygen Cylinder | 57.6  (51.4-63.5) | 81.8  (76.8-85.9) | 70.2  (66.2-73.9) | 98.6  (96.8-99.4) | 98.8  (96.9-99.6) |
| Pulse Oximeter | 19.8  (15.4-25.2) | 35.0  (29.6-40.8) | 27.7  (24.1-31.7) | 74.7  (70.3-78.7) | 94.0  (90.9-96.1) |
| Stethoscope | 99.2  (96.9-99.8) | 99.3  (97.2-99.8) | 99.3  (98.0-99.7) | 99.5  (98.1-99.9) | 100.0  (100.0-100.0) |
| **Diabetes Mellitus** | | | | | |
| Glucometer | 80.5  (75.2-84.9) | 81.8  (76.8-85.9) | 81.2  (77.7-84.3) | 93.5  (90.7-95.5) | 93.7  (90.6-95.9) |
| Biochemical Analyzer | 26.5  (21.4-32.2) | 21.4  (17.0-26.6) | 23.8  (20.4-27.6) | 61.2  (56.4-65.8) | 93.1  (89.9-95.4) |
| Reagents/ kits for Glucose test | 28.8  (23.6-34.6) | 24.3  (19.6-29.7) | 26.4  (22.9-30.4) | 57.3  (52.5-62.0) | 80.9  (76.3-84.8) |
| Lancets | 81.3  (76.1-85.6) | 81.4  (76.4-85.6) | 81.4  (77.8-84.5) | 87.2  (83.7-90.1) | 89.3  (85.5-92.2) |
| Glucostrips | 69.3  (63.3-74.6) | 70.7  (65.1-75.8) | 70.0  (66.0-73.8) | 82.4  (78.4-85.8) | 89.6  (85.8-92.4) |
| Urine strips (Protein) | 59.5  (53.4-65.4) | 66.1  (60.3-71.4) | 62.9  (58.8-66.9) | 86.5  (82.9-89.5) | 94.9  (92.0-96.8) |
| **Miscellaneous** | | | | | |
| Infusion set | 62.6  (56.5-68.4) | 75.7  (70.3-80.4) | 69.5  (65.4-73.2) | 91.8  (88.7-94.1) | 94.9  (92.0-96.8) |
| Laryngoscope | 18.7  (14.4-23.9) | 29.6  (24.6-35.3) | 24.4  (20.9-28.2) | 71.6  (67.0-75.7) | 88.7  (84.8-91.6) |
| Adult ambu bag | 38.5  (32.8-44.6) | 72.1  (66.6-77.1) | 56.1  (51.8-60.2) | 90.6  (87.4-93.1) | 96.1  (93.4-97.7) |
| Torch/ Examination light | 83.7  (78.6-87.7) | 84.3  (79.5-88.1) | 84.0  (80.6-86.9) | 88.9  (85.5-91.6) | 91.6  (88.2-94.2) |
| Vaginal Speculum | 63.0  (56.9-68.7) | 75.7  (70.3-80.4) | 69.6  (65.6-73.4) | 90.4  (87.1-92.9) | 83.6  (79.2-87.2) |
| Microscope | 73.9  (68.2-78.9) | 77.9  (72.6-82.4) | 76.0  (72.2-79.4) | 97.1  (95.0-98.4) | 99.7  (97.9-100.0) |
| X-Ray Machine | NA | NA | NA | 72.5  (68.0-76.6) | 98.8  (96.9-99.6) |
| Ultrasound | NA | NA | NA | 38.6  (34.0-43.3) | 94.3  (91.3-96.4) |
| CAT Scan | NA | NA | NA | 4.1 (2.6-6.5) | 48.1  (42.7-53.4) |

* NA- Not available
