# Supplementary material for: Preparedness of primary and secondary health facilities in India to address major noncommunicable diseases: results of a National Noncommunicable Disease Monitoring Survey (NNMS)
Source: BMC Health Serv Res. 2021 Jul 31;21:757. doi: 10.1186/s12913-021-06530-0 (PMC8325187; doi:10.1186/s12913-021-06530-0)
Supplement: Supplementary file 3 — Additional file 3: Additional Table 3. Availability (%) of technical human resources in public primary urban and rural health facilities in India; NNMS (2017–18). [file 12913_2021_6530_MOESM3_ESM.docx]

Additional table 3: Availability (%) of technical human resources in public primary urban and rural health facilities in India; NNMS (2017-18)

| **Staff Category** | **Primary public health care facilities (n =537)** | | | |
| --- | --- | --- | --- | --- |
|  | **Urban**  **N=257** | | **Rural**  **N=280** | |
|  | % (95% CI) of Health facility where mentioned staff is | | | |
|  | **Available** | **Trained** | **Available** | **Trained** |
| **Doctors** |  | | | |
| **General Duty Medical Officers** | | | | |
| Allopathic System | 84.8  (79.9-88.7) | 32.7  (27.2-38.7) | 85.4  (80.7-89.0) | 28.2  (23.2-33.8) |
| AYUSH system | 23.0  (18.2-28.5) | 2.3  (1.0-5.1) | 35.0  (29.6-40.8) | 7.9  (5.2-11.7) |
| **Specialist Medical Officers** | | | | |
| Medicine | 14.8  (10.9-19.7) | 4.3  (2.4-7.6) | 7.1  (4.6-10.8) | 2.5  (1.2-5.2) |
| Surgery | 2.7  (1.3-5.6) | NA | 3.6  (1.9-6.5) | NA |
| Obs & Gynae | 18.7  (14.4-23.9) | 3.5  (1.8-6.6) | 6.4  (4.1-10.0) | 0.4  (0.0-2.5) |
| Ophthalmologist | 10.9  (7.6-15.3) | 1.2  (0.4-3.6) | 5.4  (3.2-8.7) | 1.4  (0.5-3.8) |
| **Nursing and paramedical Staff** | | | | |
| Staff Nurse | 73.5  (67.8-78.6) | 22.6  (17.9-28.1) | 67.5  (61.8-72.7) | 24.3  (19.6-29.7) |
| Health Assistants or equivalent | 40.1  (34.2-46.2) | 9.7  (6.6-14.0) | 51.8  (45.9-57.6) | 12.1  (8.8-16.5) |
| Auxiliary Nurse Midwife or equivalent | 70.0  (64.1-75.3) | 15.2  (11.3-20.1) | 76.4  (71.1-81.0) | 17.9  (13.8-22.8) |
| Pharmacist | 82.9  (77.8-87.0) | 14.8  (10.9-19.7) | 81.1  (76.0-85.3) | 14.3  (10.6-18.9) |
| Lab Technician | 73.5  (67.8-78.6) | 13.6  (9.9-18.4) | 68.2  (62.5-73.4) | 15.0  (11.3-19.7) |
| Physiotherapist | 2.3  (1.0-5.1) | 0.0 | 0.7  (0.2-2.8) | 0.0 |
| Care coordinator | 2.3  (1.0-5.1) | 1.2  (0.4-3.6) | 3.9  (2.2-7.0) | 0.7  (0.2-2.8) |
| Counsellor | 6.6  (4.1-10.4) | 1.2  (0.4-3.6) | 10.4  (7.3-14.5) | 3.9  (2.2-7.0) |
