# Supplementary material for: Preparedness of primary and secondary health facilities in India to address major noncommunicable diseases: results of a National Noncommunicable Disease Monitoring Survey (NNMS)
Source: BMC Health Serv Res. 2021 Jul 31;21:757. doi: 10.1186/s12913-021-06530-0 (PMC8325187; doi:10.1186/s12913-021-06530-0)
Supplement: Supplementary file 4 — Additional file 4: Additional Table 4. Noncommunicable diseases (NCD) related services being provided in the public primary urban and rural study facilities; NNMS (2017–18). [file 12913_2021_6530_MOESM4_ESM.docx]

Additional table 4: Noncommunicable diseases (NCD) related services being provided in the public primary urban and rural study facilities; NNMS (2017-18)

|  | **Public Primary health care facilities** | |
| --- | --- | --- |
|  | **Urban**  **N=257** | **Rural**  **N=280** |
| **NCD Services** | % (95% CI) | |
| Ambulatory care | 63.8  (57.7-69.5) | 77.1  (71.8-81.7) |
| In-patient care | 35.0  (29.4-41.1) | 67.5  (61.8-72.7) |
| Emergency care | 34.2  (28.7-40.3) | 46.4  (40.6-52.3) |
| Screening for NCDs | 89.5  (85.1-92.7) | 86.4  (81.9-90.0) |
| Counselling for NCDs | 17.5  (13.3-22.7) | 13.2  (9.7-17.7) |
| Physiotherapy | 4.7  (2.7-8.1) | 2.9  (1.4-5.6) |
| Laboratory testing for major NCDs | 68.1  (62.1-73.5) | 70.4  (64.7-75.4) |
| Availability of management guidelines in the hospital | 49.0  (42.9-55.1) | 26.1  (21.3-31.5) |
| Display of NCD related IEC materials inside the hospital | 69.3  (63.3-74.6) | 57.1  (51.3-62.8) |
| **Median (IQR) NCD patient load per month** | | |
| New Out-patients | 103 (302) | 61(227) |
| NCD admissions | 0 (10) | 0 (7) |
